# Direct Light-up of cAMP Derivatives in Living Cells by Click Reactions

**DOI:** 10.3390/molecules181012909

**Published:** 2013-10-17

**Authors:** Kenichiro Ito, Hongshan Liu, Makoto Komiyama, Tetsuya Hayashi, Yan Xu

**Affiliations:** 1 Research Center for Advanced Science and Technology, The University of Tokyo 4-6-1 Komaba, Meguro-ku, Tokyo 153-8904, Japan; 2 Division of Chemistry, Department of Medical Sciences Faculty of Medicine, University of Miyazaki 5200 Kihara, Kiyotake, Miyazaki 889-1692, Japan; 3 Division of Microbial Genomics, Department of Genomics and Bioenvironmental Science, Frontier Science Research Center, and Division of Microbiology, Department of Infectious Diseases, Faculty of Medicine, University of Miyazaki, 5200 Kiyotake, Miyazaki 889-1692, Japan

**Keywords:** click chemistry, cAMP, fluorescence, cAMP localization in living cells

## Abstract

8-Azidoadenosine 3′,5′-cyclic monophosphate (8-azido cAMP) was directly detected in living cells, by applying Cu-free azide-alkyne cycloaddition to probe cAMP derivatives by fluorescence light-up. Fluorescence emission was generated by two non-fluorescent molecules, 8-azido cAMP as a model target and difluorinated cyclooctyne (DIFO) reagent as a probe. The azide-alkyne cycloaddition reaction between 8-azido cAMP and DIFO induces fluorescence in 8-azido cAMP. The fluorescence emission serves as a way to probe 8-azido cAMP in cells.

## 1. Introduction

3′,5′-Cyclic adenosine monophosphate (cAMP) is one of the most studied second messenger molecules that governs many fundamental cellular functions and processes, including metabolism, gene expression, and cell division [1,2,3,4]. To understand the cAMP-dependent signaling mechanisms, two classes of sensors that detect cAMP as a target molecule have been developed [5,6,7,8,9]. One is based on protein kinase A (PKA), using fluorescence as an indicator to detect cAMP localization [5,6,7,8,9], and the other exploits the exchange protein directly activated by cAMP (EPAC) [5,6,7,8,9]. Both of these sensors utilize heterologously expressed effector proteins to measure cAMP. The two sensors do not directly detect cAMP, but rather depend on protein expression mediated by cAMP. This creates an unavoidable problem in terms of a lack of sensitivity. For instance, a major problem is that the quantity of protein-based sensors is dependent on the expression level of each cell. Another problem is that strategies involving these two sensors need to construct complicated plasmids of fusion proteins as indicators. Thus, a method for direct detection of cAMP in living cells will offer the greater convenience of a simpler strategy and give a more accurate result in terms of cAMP detection.

In this study, we describe the application of the azide–alkyne cycloaddition [10,11] the most extensively studied “click reaction”, to probe cAMP derivatives in living cells. Click chemistry has previously been used to functionalize various biological materials, such as membranes [12], proteins [13], and oligonucleotides [14,15], as well as to probe DNA and RNA G-quadruplex structures in our laboratory [16,17]. Bertozzi *et al.* recently reported a difluorinated cyclooctyne (DIFO) reagent that rapidly reacts with azides in living cells without the need for copper catalysis [18,19,20]. Using the DIFO probe, we can rapidly and sensitively detect cAMP derivatives by fluorescence light-up in living cells without the need for the usual fixing and washing steps.

## 2. Results and Discussion

**Figure 1:**
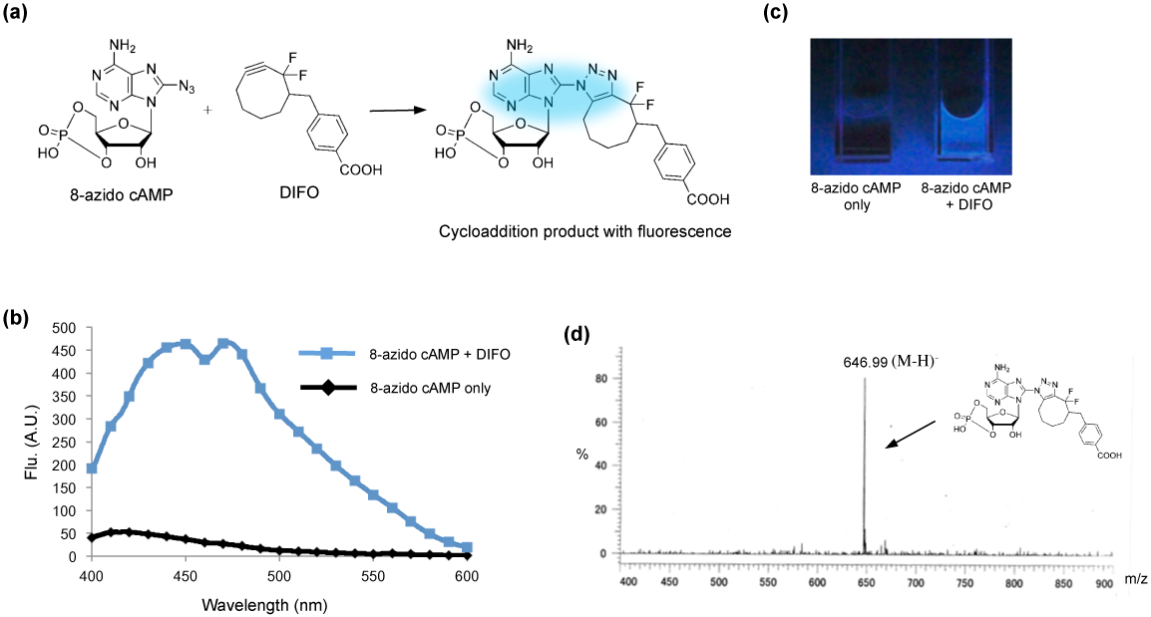
Azide-alkyne cycloaddition in Cu-free conditions. (**a**) Two molecules, 8-azidoadenosine 3′,5′-cyclic monophosphate (8-azido cAMP) and a DIFO reagent, were conjugated by Cu-free azide-alkyne cycloaddition. The Cu-free reaction adds a triazole ring at the 8-position of 8-azido cAMP, resulting in a nucleoside analog that shows fluorescence. (**b**) Fluorescence spectra for the cycloaddition reaction product and 8-azido cAMP. Reaction conditions: [8-azido cAMP] = 1 mM, [DIFO] = 1 mM, 37 °C, 5 h. (**c**) Fluorescence image of the cycloaddition reaction product (right) and 8-azido cAMP (left) after illumination with a UV lamp (365 nm). (**d**) ESI-MS spectrum of the cycloaddition reaction product.

We used 8-azidoadenosine 3′,5′-cyclic monophosphate (8-azido cAMP) as the model target of detection. It has been suggested that functionalizing the adenosine derivatives results in nucleoside analogs with fluorescence properties [21,22,23,24]. Before the cycloaddition reaction, 8-azido cAMP and the DIFO reagent display no fluorescence with 365-nm excitation (Figure 1a). Formation of a triazole ring in the 8-position by azide-alkyne cycloaddition induces a strong fluorescence in 8-azido cAMP (Figure 1a). While the normal 8-azido cAMP nucleoside has no fluorescence, the triazole adduct acts as a fluorophore and emits at 400–500 nm (Figure 1b). A clear blue emission could be observed by the naked eye upon UV excitation of a 100 μL, 1:1 molar ratio mixture of 8-azido cAMP and the DIFO reagent (Figure 1c). In contrast, 8-azido cAMP solution alone did not show any fluorescence under the same UV excitation (Figure 1c). To further characterize the cycloaddition product, electrospray ionization mass spectrometry (ESI-MS) was used to directly observe the cAMP-derivative formation. We observed a peak near m/z 646.99 (M-H)^−^, corresponding to the molecular weight of product 1 (MW = 648.17) (Figure 1d), suggesting that azide-alkyne cycloaddition occurs under Cu-free conditions. These results suggest that the DIFO reagent can directly recognize cAMP by azide-alkyne cycloaddition with emission of blue fluorescence. Furthermore, the DIFO reagent can directly react with 8-azido cAMP by azide-alkyne cycloaddition resulting in a nucleoside analog that shows fluorescence. The advantage of the reaction dependence of an emission production is that the procedure does not require extensive washing steps to remove the unreacted fluorescent dyes in the cell. The benefit of direct light-up of cAMP may allow the approach more useful than another cAMP analogue, 8-N3-6-[DY-547]-cAMP, which is a fluorescent cAMP analog [25]. The DY-547 self-probe with a larger molecular size than 8-azido cAMP will create steric hindrance to influence the interaction between the cAMP analogue and the associated proteins.

**Figure 2:**
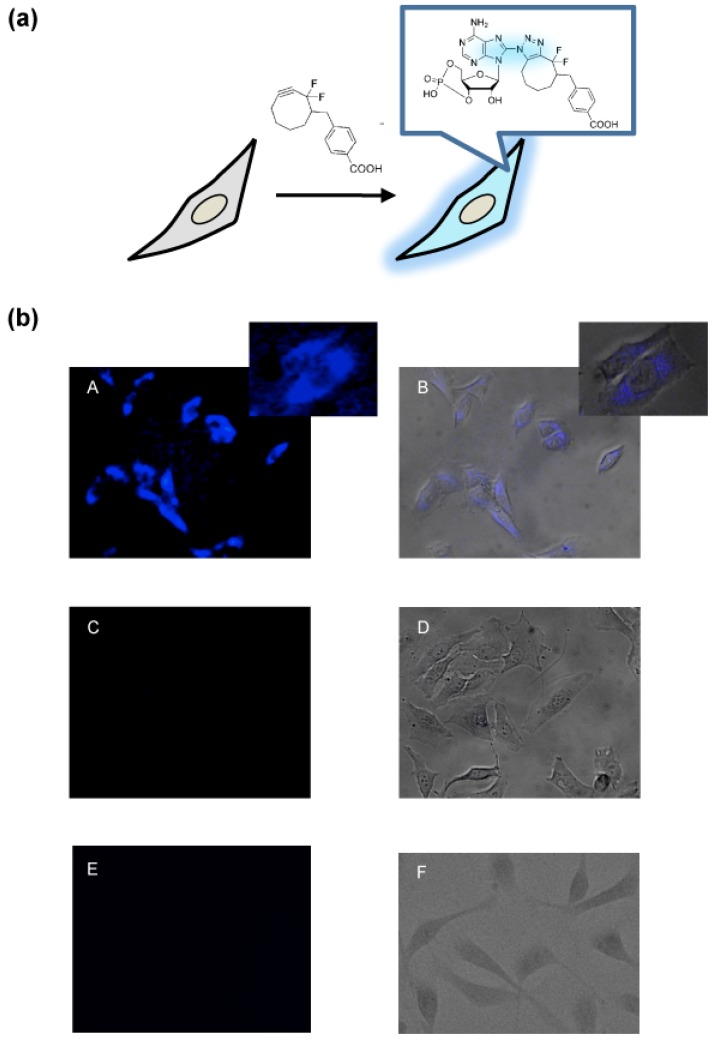
Direct detection of cAMP in living cells. (**a**) Schematic for detection of cAMP in living cells using the DIFO reagent. The azide-alkyne cycloaddition product in living cells emits fluorescence. (**b**) Fluorescence microscopy images of live cells. (A, B) Hela cells with 8-azido cAMP were incubated with the DIFO reagent. Small panels show the enlarged image. (C, D) Hela cells without 8-azido cAMP as a negative control. (E, F) Hela cells without the DIFO reagent as a negative control. B, D and F panels show fluorescence images merged with bright field images.

**Figure 3:**
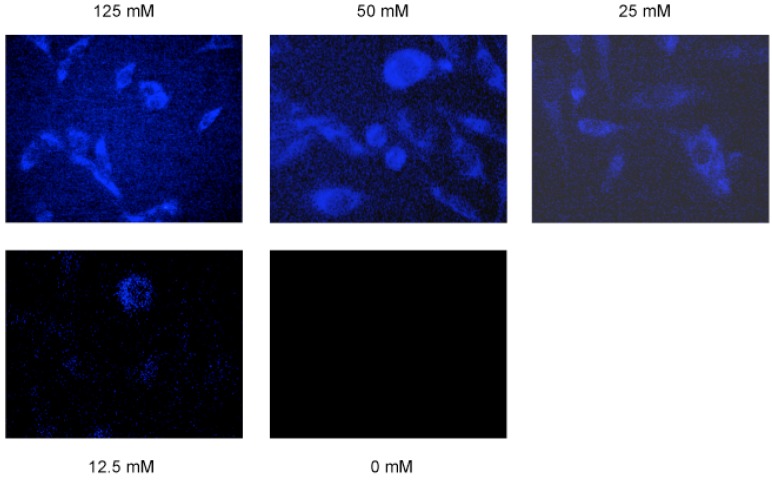
8-Azido cAMP was detected by cycloaddition reaction with various concentration of DIFO.

Once an efficient cycloaddition reaction was established, we tested whether the DIFO reagent was able to detect cAMP derivative in living cells (Figure 2a). HeLa cells were incubated for 3 h in a CO_2_ incubator after the transfection of 8-azido cAMP. The cycloaddition reaction was initiated by adding a new cell medium containing 125 μM of the DIFO reagent. Without the cellular fixing and washing steps, we were able to observe the cells directly by use of a fluorescent microscope with 365 nm UV irradiation. We clearly observed the blue fluorescence from the DIFO-treated cells with a 470/40 nm filter (Figure 2b). In the negative control cells without 8-azido cAMP or the DIFO reagent, it was almost impossible to find the backgroud fluorescence (Figure 2b). Next, we performed a concentration-dependent imaging experiment in living cells. We found that the fluorescence intensity increased with increasing concentration of the DIFO reagent (Figure 3).

## 3. Experimental

### General

8-Azidoadenosine 3′,5′-cyclic monophosphate (8-azido cAMP) was purchased from Sigma Aldrich (Tokyo, Japan). The UV irradiation (300 < λ < 400 nm) was achieved with a UV Spot Light Source (200 W, Hamamatsu Photonics, Hamamatsu, Japan) and a UV-D36C filter (Asahi Technoglass, Sizuoka, Japan) at 0.1 W/cm^−2^min^−1^. Fluorescent spectra were measured using a Jasco model FP-6500 spectrofluorometer (Jasco, Tokyo, Japan). ESI-MS spectra were measured with an M-8000 Mass Spectrometer (Hitachi, Tokyo, Japan). Cells were observed with an AF-6000 microscope (Leica Microsystems, Tokyo, Japan). The fluorescent spectra were recorded using a 1 cm path length cell and 365 nm excitation. 8-azido cAMP and the DIFO reagent were mixed at 1:1 molar ratio (0.2 mM) and then incubated at 37 °C for 5 h. Hela cells (5 × 10^5^ cells/mL) were transfected using a Neon^TM^ Transfection System (Invitrongen, Carlsbad, CA, USA) and 1 mM 8-azido cAMP with 1,050 V pulse voltge, 35 ms pulse width, and 2 times pulse number. Then the cells were seeded in a 96-well tissue cluture plate and incubated at 37 °C for 3 h in Dulbecco’s modified Eagle’s medium (DMEM, 1.5 ml) containing 10% fetal bovine serum (FBS). The DIFO reagent (0–125 μM) was added to the medium and incubated at 37 °C for 14 h. The cells were washed three times with a buffer solution (PBS; 140 mM NaCl, 2.7 mM KCl, 10 mM Na_2_HPO_4_, 1.8 mM KH_2_PO_4_, pH 7.3). The excitation and absorbance filters were 360/40 and 470/40 nm, respectively. In the negative control cells without the DIFO reagent, 8-azido cAMP was transfected into cell by Lipofectamine 2000 reagent (Invitrogen), and cells were observed with a BZ-9000 microscope (Keyence Corporation, Osaka, Japan).

## 4. Conclusions

In summary, we have described herein the first successful imaging of a cAMP derivative in living cells by azide-alkyne cycloaddition. The Cu-free reaction results in a nucleoside analog with fluorescence properties. The direct light-up of cAMP makes the cellular fixing and washing steps unnecessary. In contrast to previous reports using the expressed effector proteins to measure cAMP, the click chemistry strategy directly targets cAMP and does not depend on intracellular environmental factors. In addition, the approach may allow for detection of the specific intracellular site of cAMP derivatives. Moreover, the method will enable the measurement of cAMP fluctuations as they happen in the complex intracellular environment and are proving to be effective tools to probe the specific signaling complexes within cells.
